# Embryonic Leucine Promotes Early Postnatal Growth via mTOR Signalling in Japanese Quails

**DOI:** 10.3390/ani14172596

**Published:** 2024-09-06

**Authors:** Sawadi F. Ndunguru, Gebrehaweria K. Reda, Brigitta Csernus, Renáta Knop, James K. Lugata, Csaba Szabó, Ádám Z. Lendvai, Levente Czeglédi

**Affiliations:** 1 Department of Animal Science, Institute of Animal Science, Biotechnology and Nature Conservation, Faculty of Agricultural and Food Sciences and Environmental Management, University of Debrecen, 4032 Debrecen, Hungary; sawadindunguru@gmail.com (S.F.N.); gebrek2000@gmail.com (G.K.R.); dr.knop.renata@agr.unideb.hu (R.K.); czegledi@agr.unideb.hu (L.C.); 2 Doctoral School of Animal Science, University of Debrecen, 4032 Debrecen, Hungary; jkachungwa@gmail.com; 3 Department of Evolutionary Zoology and Human Biology, University of Debrecen, 4032 Debrecen, Hungary; csernusb@science.unideb.hu (B.C.); az.lendvai@gmail.com (Á.Z.L.); 4 Department of Animal Nutrition and Physiology, Institute of Animal Science, Biotechnology and Nature Conservation, Faculty of Agriculture and Food Sciences and Environmental Management, University of Debrecen, 4032 Debrecen, Hungary

**Keywords:** birds, physiology, nutrition, amino acid, mechanistic target of rapamycin

## Abstract

**Simple Summary:**

Nutritional cues during embryonic development significantly impact growth, although the mechanism behind this influence remains unclear. Amino acids such as leucine can affect the nutrient-sensing pathway that regulates growth. We injected 2.5 mg of leucine or saline into Japanese quail eggs on the tenth day of incubation. Treatment groups showed no significant difference in hatching success, body mass, gastrointestinal length, and morphological traits (wing, tarsal, and head lengths). However, from day 3 to day 7 post-hatch, chicks hatching from leucine-treated eggs showed increased wing length, body mass, tarsal length, and head and intestinal lengths, which lasted up to 21 days. Similarly, the growth-related genes in the liver were upregulated in leucine-treated quail chicks. However, protein degradation genes remained unchanged. These results suggest that the slight increase in embryonic leucine can promote growth, highlighting the potential for improvement in poultry growth performance.

**Abstract:**

Nutritional cues during embryonic development can alter developmental trajectories and affect postnatal growth. However, the specific mechanisms by which nutrients influence avian growth remain largely unknown. Amino acids can directly interact with the nutrient-sensing pathways, such as the insulin-like growth factor 1 (IGF-1)/mechanistic target of rapamycin (mTOR) pathways, which are known to regulate growth. We examined the effects of embryonic leucine on gene expression and phenotypic growth in Japanese quails by injecting 2.5 mg leucine or saline (control) into Japanese quail eggs on the tenth day of incubation and incubating them under standard conditions. The treatment groups had similar hatching success and size at hatching. However, between 3 and 7 days post-hatching, quails treated with embryonic leucine showed increased growth in body mass and wing, tarsus, head, and intestinal lengths, lasting up to 21 days. The hepatic expression of *IGF1*, *IGF1R*, *mTOR*, and *RPS6K1* was upregulated in leucine-treated quails, while the expression of *FOXO1* remained unaffected. In conclusion, a subtle increase in embryonic leucine may induce developmental programming effects in Japanese quail by interacting with the IGF-1/mTOR nutrient-sensing pathway to promote growth. This study highlights the role of embryonic amino acids as crucial nutrients for enhancing growth. It provides valuable insight into nutrient intervention strategies during embryonic development to potentially improve poultry growth performance.

## 1. Introduction

Nutritional programming during early life profoundly affects phenotype. Early nutritional supplementation may boost postnatal growth and development, which can have long-lasting effects [1,2,3]. Conversely, early poor nutrition has enduring adverse effects [4]. Recent studies have shown the importance of prenatal nutrients over traditional postnatal focus, highlighting their potential and impacts [5,6]. Understanding the mechanisms through which specific nutrients influence phenotypic expression in birds, mainly through embryonic programming, is crucial and still emerging [5,7]. While several nutrients may influence development, maternally and externally derived amino acids have been recognised to have the potential to alter the developmental trajectory and thus have phenotypic programming effects [8,9].

Amino acids exert their developmental effects through two connected nutrient-sensing pathways: the insulin/insulin-like factor 1 signalling (IIS) and the mechanistic target of rapamycin (mTOR) [10,11]. Insulin-like growth factor 1 (IGF-1), primarily secreted from the liver, is a crucial regulator of energy metabolism, protein synthesis, and cellular proliferation, affecting growth, reproduction, and lifespan in response to energy and nutrient status [12]. Changes in nutritional status stimulate the growth hormone GH/*IGF-1* axis, leading to the alteration of IGF-1 levels [13,14]. The effects of IGF-1 are facilitated through the IGF-1 receptor (IGF-1R), which interacts with the mTOR complex network of intracellular nutrient pathways in influencing growth [15,16]. Growth is enhanced when the protein synthesis rate surpasses the protein degradation rate regulated by pathways involved with the protein kinase B or Akt, which acts as a critical regulator, playing a significant role in both protein synthesis and degradation in the nutrient-sensing pathways [17,18]. This kinase increases the activity of mTOR but negatively regulates the forkhead box protein O1 (FOXO1) transcription factor, thereby reducing protein degradation [18,19].

Recently, we have shown that an experimental increase in embryonic methionine, an essential amino acid, increased the growth and development of Japanese quail chicks, which resulted in higher body mass from 5 days to 21 days post-hatch [8]. The increase in postnatal growth in quail chicks was associated with the activation of the nutrient-sensing pathways, triggering the upregulation of hepatic *IGF1* and *mTOR* genes one day postnatal, while the expression of mTOR’s downstream effector, ribosomal protein serine 6 kinase 1 (*RPS6K1*), and circulating IGF-1 followed 21 days post-hatching [8]. These results provided further room for investigating how other specific amino acids influence growth via the modulation of the IGF-1/mTOR system as different amino acids operate through distinct mechanisms [20,21]. Building our understanding on methionine, we now explore the role of leucine, another essential amino acid. 

Leucine, a branched-chain essential amino acid (BCAA), has a critical role in development through its effects on protein synthesis and metabolism. Insufficient dietary leucine impairs growth [22], while excessive dietary leucine supplementation above the recommended level does not improve weight gain and growth in broiler chicken [18,23]. Increased dietary leucine in rats and early-life broiler chicks improves protein synthesis and muscle maintenance through *mTOR* activation and also affects *RPS6K1* expression in the muscles [24,25]. However, not all studies show that leucine boosts growth, indicating that its effects may depend on specific conditions [18]. 

Therefore, we hypothesised that embryonic leucine could influence the hormonal and gene expression patterns in the nutrient-sensing pathways that influence postnatal growth and development in Japanese quails (*Coturnix japonica*), a model species frequently used for exploring the influence of early-life conditions or embryonic development [26]. Thus, we determined the hepatic expression of growth-related genes (*IGF1*, *IGF1R*, *mTOR*, *RPS6K1*) and the transcription factor for autophagic genes *FOXO*1 to study the influence of in ovo application of leucine on early growth and development. 

## 2. Materials and Methods

### 2.1. Experimental Animals and Analysis of Amino Acid Concentration in Eggs

Experiments were approved by the Institutional Committee of Animal Welfare permit number 5/2021/DEMAB. We carried out the trial at the experimental farm of the University of Debrecen, Farm and Regional Research Institute, Kismacs, Hungary. We collected 336 eggs from 70 laying Japanese quails (*Coturnix japonica*), 33 weeks old, over five days and kept them between 16 °C and 18 °C. We weighed the eggs using a digital scale (0.01 g accuracy). We selected eggs with similar mass (11.0 ± 0.5 g, 100 eggs) to reduce the effects of egg mass differences [8].

To determine the natural range of amino acids in freshly laid eggs, we quantified amino acid concentrations from a pool of 12 eggs at the accredited Central Laboratory of the Agriculture and Food Products, Faculty of Agricultural and Food Sciences and Environmental Management University of Debrecen, Hungary. The protein content of the samples was determined using the Kjeldahl method [27]. First, the nitrogen content of the sample was converted into ammonium salt by boiling it in concentrated sulphuric acid. A total of 14 mL of concentrated sulphuric acid and two catalyst tablets were added containing selenium. The sample was destructed at 420 °C when placed on a destructive block (VELP DKL Kjeldahl, VELP Scientifica Srl, Usmate, Italy). After cooling the sample, it was distilled on a VELP UDK-149 (VELP Scientifica Srl, Usmate, Italy) distiller. An automatic titrator (VELP TITROLINE 5000, VELP Scientifica Srl, Usmate, Italy) was applied, and the nitrogen content was calculated. The protein content of the samples was calculated from the nitrogen content using a conversion factor (6.25). Measurements were repeated four times with CV% < 10%.

For protein hydrolysis, the same amount of protein was measured into a hydrolysis tube with Teflon top with 6N HCl and was reacted at 110 °C for 23 h in an oven (Memmert UN55, Buechenbach, Germany). The samples were applied to the amino acid analysis after cooling and filtering through a regenerated cellulose filter (0.2 µm, Whatman 10463040 Spartan syringe filter, Cytiva, Marlborough, MA, USA). For total amino acid analysis, an AAA500 amino acid analyser (INGOS Ltd., Prague, Czech Republic) with low-pressure ion-exchange chromatography with post-column derivatisation with ninhydrin (INGOS Ltd., Prague, Czech Republic) and photometric detection at 210 and 254 nm was used. An amino acid standard mixture (INGOS Ltd., Prague, Czech Republic) was applied as a reference. The recovery was higher than 95%. Results are reported in Table 1.

### 2.2. Incubation and in Ovo Injection of Leucine 

We incubated 100 eggs in an automatic turning incubator (WQ-63-WQ-98 Model 2021 Version 2, AGROFORTEL, SRO, Budapest, Hungary). On day 10 of the embryonic development, we candled the eggs and removed those containing dead embryos and unfertilised eggs from the incubator. We set the incubation temperature at 37.8 ± 0.5 °C and relative humidity at 50–60%. 

We prepared the leucine solution (50 mg leucine/mL saline solution) by dissolving crystalline L-leucine (reagent grade, purity > 98%; Sigma Aldrich, Merck Life Science Ltd., Budapest, Hungary) in a 0.9% physiological saline solution (B. Braun Melsungen AG, Melsungen, Germany). Saline solution serves a neutral baseline for comparison to the effects of the active or real treatment group. After disinfecting the eggshell with 70% ethanol, we incised it using a sterile 26G needle through the broad end site of the eggs. We injected 39 randomly selected eggs (50 μL into each egg) with 2.5 mg/egg of the leucine solution into the amniotic fluid using a Hamilton syringe on embryonic day ten (ED10). Then, the second group of 36 eggs received 50 μL of the physiological saline solution (B. Braun Melsungen AG, Melsungen, Germany) only, each serving as a control group. We used a completely randomised experimental design where eggs with similar masses were allocated randomly to the two treatments. After the injection, we sealed the hole with candle wax and transferred the egg to the incubator to resume incubation. Based on the average leucine content of eggs (Table 1) and the average mass of the eggs (11.0 g), the injection represents an average 2.0% increase in leucine content.

### 2.3. Rearing Experimental Hatchlings and Sample Collection 

On day 14 of the incubation, we transferred the eggs from the incubator tray to the partitioned hatching tray. The hatching tray was portioned based on the treatment groups to separate the eggs and avoid mixing of chicks during hatching. We reduced the incubation temperature to 35.5 °C, and relative humidity increased to 65–70%. Hatching of the chicks was inspected twice daily. A total of 24 chicks hatched from leucine-treated eggs and 18 chicks from the control group. Hatchlings from each experimental group were tagged with numbered rings and immediately transferred to cages (40 cm long × 50 cm wide × 40 cm height) and reared for an average of three weeks (21 days) in groups of their treatments. We gave the hatchlings free access to water and feed (Table 2). We kept chicks under uniform standard conditions throughout the experimental period. The rearing temperature was 37 °C at the beginning up to four days provided by infrared lamps, then reduced by 3 °C every four days until reaching 22 °C by the end of the third week. The cage’s relative humidity was between 60–65%, while light was provided 24 h up to the age of 21 days. We recorded post-hatch body mass using an electronic scale with a precision of 0.01 g, VWR software version 6.02 (Avantor, Radnor, PA, USA). We also measured wing, tarsus, and head lengths using a vernier calliper (to the nearest 0.01 mm) on days 1, 3, 5, 7, 14, and 21 post-hatching. Although we did not perform a subjective analysis, body mass and morphological measurements were taken by the same person without knowing the treatment, and we used sample codes during the laboratory analyses. After recording body mass, we randomly selected eight (8) chicks from each experimental group for sample collection on day-old and 21-day-old birds. At the end of the experimental period, we sacrificed birds by cervical dislocation. We measured the whole gastrointestinal tract (GIT) length post-mortem on day-old and 21-day-old chicks using a ruler (nearest 0.1 mm). Changes in the size of the gastrointestinal tract reflect how nutrient supplements affect the physical growth of the digestive system, which is vital for efficient nutrient absorption and the overall development of chicks. Then, liver samples were collected, snap-frozen in dry ice, and stored at −80 °C for further gene expression assay.

### 2.4. RNA Isolation and Real-Time qPCR

We excised about 25 mg of the frozen liver tissue, homogenised it in 700 μL TRK lysis buffer, and isolated the total RNA using peqGold Total RNA kit following the manufacturer’s protocol (VWR International LLC., Radnor, PA, USA). The peqGOLD DNase digestion kit was used for RNA isolation. We measured the total RNA concentration spectrophotometrically at a 260 nm wavelength using an HTX Synergy Multi-Mode Microplate Reader (Agilent BioTek, BioTek Instrument Inc., Winooski, VT, USA). The ratio of the absorbance at 260/280 and 260/230 for protein purity and organic solvent purity of the samples ranged from 1.8 to 2.0 and was considered appropriate. We assessed the RNA integrity using QubitTM IQ Assay with values ranging from 8.4–9.2 scores using the manufacturer’s protocol (Life Technologies Corporation, ThermoFisher Scientific, Bleiswijk, The Netherlands). 

We used 200 ng for the reverse transcription based on the total RNA concentration. We synthesised cDNA using the LunaScript^®^ RT SuperMix Kits kit following the manufacturer’s instructions (New England Biolabs Inc., Ipswich, MA, USA) in a PCRmax Alpha Thermal Cycler (Cole-Parmer Ltd., Vernon Hills, IL, USA). The LunaScript^®^ SuperMix reaction mix contained a random hexamer and oligo-dT primers, dNTPs, Murine RNase Inhibitor, and Luna^®^ Reverse Transcriptase. The total reaction mix volume was 20 µL with the reaction mixture of nuclease-free water, 4 µL LunaScript^®^ SuperMix, and RNA template. The conditions cycles for cDNA synthesis were 2 min at 25 °C for primer annealing, 10 min at 55 °C for cDNA synthesis, 5 min at 95° C for heat activation, and holding at 4 °C. cDNA samples were diluted 10-fold and stored at −20 °C.

We designed quail-specific, intron-spanning primers from Integrated DNA Technologies (BVBA-Leuven, Belgium) using the Oligo 7 software (Table 3). We verified the primer target specificity using Primer-Blast web based tool (https://www.ncbi.nlm.nih.gov/tools/primer-blast/, accessed on 20 September 2021) based on the mRNA nucleotide sequence of the goal gene from the National Center for Biotechnology Information (NCBI, http://www.ncbi.nlm.nih.gov, accessed on 20 September 2021).

We performed a quantitative polymerase chain reaction (qPCR) for cDNA amplification using EvaGreen qPCR Mix according to the manufacturer’s protocol (Solis BioDyne, Teaduspargi, Estonia). The total reaction volume was 10 µL, with the reaction mixture of nuclease-free water, 200 nM of each primer, 5 × H.O.T. FIREPol^®^ EvaGreen^®^ qPCR Mix Plus (ROX) (Solis BioDyne, Tartu, Estonia) supermix, and 2 ng cDNA template. The qPCR reaction conditions were performed for 12 min at 95 °C, with initial activation of the polymerase followed by 40 cycles of denaturation for 15 s at 95 °C, annealing for 20 s minutes at 60 °C, and elongation for 20 s at 75 °C. The amplification and melting curve analysis was performed using Agilent AriaMx Real-Time PCR System instrument software version 1.8 (Agilent Technologies, Santa Clara, CA, USA). The PCR reaction mixture was performed with duplicate measurements of each sample, and the average value of the duplicate was used for further analyses. The PCR reactions generated the data in each gene’s threshold cycle (Ct) value. We normalised the Ct values for each target gene using *RPL19* as a housekeeping gene based on its stability in all treatments [28,29]. We quantified the target gene transcript’s relative gene expression as fold change compared with the reference gene (RPL19) using the 2^−ΔΔCt^ method [30]. We selected the reference gene based on NormFinder, BestKeeper, and delta Ct algorithms [28].

### 2.5. Statistical Analysis

We expected missing data from the gene expression to result from data processing and normalisation before analyses. Gene expression with low signal intensity below the threshold may fall below the detection limit in certain samples, which are considered unreliable [31]. Genes with high variability in expression were filtered out and considered missing data. Only 3.5% of the gene expression data points were excluded from the analyses. During data analyses, linear models were used, which could handle the imbalanced data design and correct for the missing values.

We performed all statistical analyses using RStudio version 4.3.3 [32]. Graphs (images) were generated using the ‘ggplot’ function provided by the ‘ggplot2’ package version 3.4.3 [33]. To determine hatchability, a chi-squared test (χ^2^) was used to test whether there is a statistically significant difference between observed and expected hatching percentages between the treatment groups. Before fitting the model, we used Akaike’s information criterion corrected for small sample sizes (AICc) to choose the best model [34]. We used linear mixed models (LMMs) to analyse the effects of treatment on body mass and morphological traits (wing, tarsal, and head lengths) across time up to three weeks of age. We considered treatment in two levels (saline and leucine) and at seven time points (days 1, 3, 5, 7, 10, 14, and 21) and their interaction as fixed factors. Since chick body mass and morphological traits were measured multiple times for three weeks, to control for the individual variation across time and the effects of repeated measurements, we included the individual bird identity as a random factor in the model [35]. We used the function ‘lmer’ from the ‘lme4’ package to define the fixed and random effect and estimate the model parameters [36]. To compute the *p*-values, we used ‘lmerTest’ package version 3.1.3 in ANOVA [37]. To compare the means in body mass and morphological variables between the treatment groups within each day (at different time points), we used estimated marginal means by the ‘emmeans’ package and compared the means using the Tukey test at a *p* < 0.05 significance level [37,38]. To analyse the effects of treatment on hepatic gene expression of *IGF1R*, *IGF1*, *mTOR*, *RPS6K1*, and *FOXO1* and intestinal length, we used linear models, with treatment and time (days) as independent factors. One-way ANOVA was used to assess the statistical significance between the treatment groups, with *p* < 0.05 considered significant.

## 3. Results

**Figure 1:**
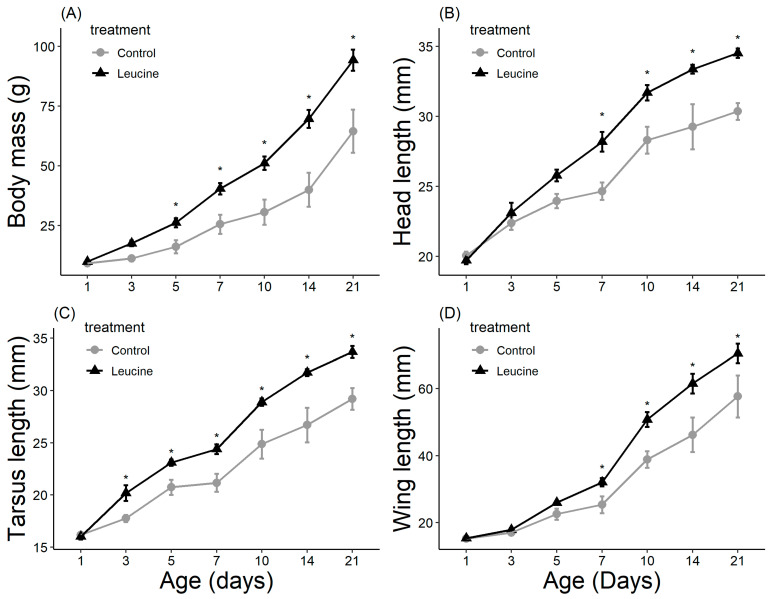
Leucine injection into Japanese quail eggs increased body mass (**A**) and head (**B**), tarsus (**C**) and wing (**D**) lengths in chicks post-hatch (see Table S1. Supplementary Materials for detailed sample size). Asterisks indicate a significant difference between the treatment groups (*p* < 0.05), and error bars indicate mean ± SE.

**Figure 2:**
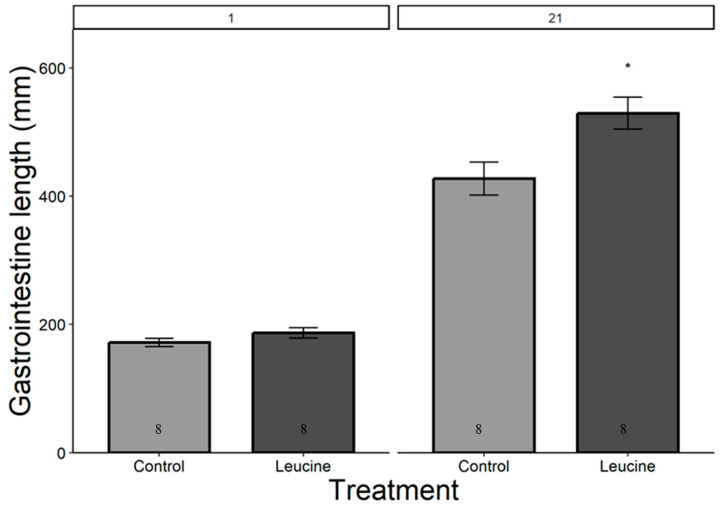
Leucine injection into Japanese quail eggs increased postnatal intestinal length in 21-day-old chicks. Numbers in the bars indicate sample size (n). The asterisk indicates a significant difference between the treatment groups at *p* < 0.05, and error bars indicate mean ± SE. Numbers 1 and 21 above the bars indicate the age of chicks in days post-hatch.

The study did not detect a statistically significant difference in hatching success between the leucine-injected (61.5%) and control (50.0%) groups (ꭓ^2^ = 0.59, df = 1, *p* = 0.440), which may be due to the sample size or the effect size being smaller than anticipated. Body mass, head length, tarsal length, and wing length were affected by both treatment, age (day), and their interactions (*p* < 0.05, for all). The treatment did not affect the chicks’ body weight at hatching (Table 4). However, starting from day 3 after hatching, chicks in the leucine injection group outgrew the controls, a difference lasting all days until day 21 (Table 4, Figure 1). At the age of 3 days, the leucine-injected group already had higher tarsus length, followed by a higher body mass at day 5, and marginally larger head length, which reached statistical significance at day 7 along with wing length, showing overall improved growth performance after embryonic leucine treatment. On day 21, birds in the leucine-treated group also had longer intestinal length than the controls (Figure 2).

**Figure 3:**
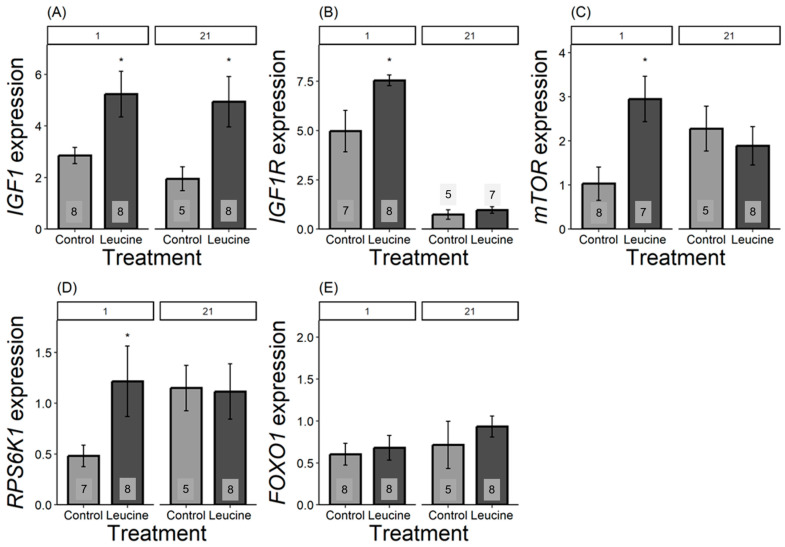
Bar plots present mRNA expression relative to the reference gene. (**A**) *IGF1*, (**B**) *IGF1R*, (**C**) *mTOR*, (**D**) *RPS6K1*, (**E**) *FOXO1* expression, light grey bars refers to control group, dark grey bars refers to leucine treatment. Numbers in the bars indicate sample size (n). The numbers at the top denote the age of the Japanese quail chicks at which the samples were collected: (1) day-old chicks and (21) 21-day-old chicks. Asterisks indicate significant differences between the treatment groups (*p* < 0.05), and error bars indicate mean ± SE.

We found that the relative expression of all genes, except for *FOXO1*, differed in one-day-old chicks and was more expressed in the leucine-treated group than in the control. For *IGF1*, the leucine-treated group had 2.5 fold higher leucine than the control, F_1,14_ = 6.42, *p* = 0.023; *IGF1R* was 1.5 fold higher following leucine treatment compared to the control, F_1,13_ = 6.31, *p* = 0.026; *mTOR* was 2 fold higher in leucine compared to the control, F_1,14_ = 9.04, *p* = 0.009; while *RPS6K1* was 0.75 fold higher in the leucine-treated group than the control, F_1,14_ = 6.53, *p* = 0.022; with no significant difference in *FOXO1*: F_1,13_ = 0.15, *p* = 0.703, Figure 3. In contrast, at day 21, only *IGF1* remained significantly more expressed in the leucine group, being 2.5 fold higher compared to the control (F_1,11_ = 5.23, *p* = 0.042; while no difference observed in *IGF1R:* F_1,9_ = 0.66, *p* = 0.435, mTOR: F_1,11_ = 0.32, *p* = 0.579; *RPS6K1*: F_1,11_ = 0.00, *p* = 0.98; *FOXO1:* F_1,11_ = 0.66, *p* = 0.432; Figure 3). 

## 4. Discussion

Hatching success was similar between the treatment and the control group, indicating that leucine treatment had no impact. These findings are consistent with previous studies where the injection of amino acids in broiler chicken eggs did not alter hatchability [39]. However, an injection of 0.2% BCAA mixture in turkey eggs had a negative impact on hatchability [5]. This contrast in hatchability suggests that factors such as species type and concentration of the amino acids can influence hatchability. 

We observed that one day after hatching, body mass and gastrointestinal, wing, tarsal, and head lengths were similar between treatment groups. Although feed intake during the postnatal period could play a prominent role in growth, it was not measured. However, chicks showed faster growth in the leucine-injected group; by day 7, they were heavier and bigger than the controls (Figure 1, Table 4). Some morphological traits already showed an advantage in the leucine-treated group by day 3 (tarsus) or day 5 (body mass), while head length and wing length only reached statistical significance by day 7. While our study used a 2.0% increase in leucine, evidence shows that embryonic feeding of a 0.1% BCAA blend including leucine before the onset of incubation increased only the embryonic growth with no difference in weight of the newly hatched chicks [40]. In contrast, an embryonic feeding of a 0.2% BCAA blend at day 22 of incubation decreased the embryo weight [5]. Still, a 0.2% BCAA blend increased the weight of freshly hatched poults in turkey by improving skeletal muscle development [5]. These contradictory results may be related to the number of amino acids injected and the species. In any case, our results, consistently with [8] showed that embryonic supplementation of amino acids can achieve long-lasting enhancement of growth performance in Japanese quails. 

Gastrointestinal growth is necessary to ensure nutrient absorption to meet the demand of chicks. The maximal growth in intestinal length usually occurs between 4 and 8 days post-hatch, with body mass increasing more than six times in chickens [41]. In our study, leucine treatment increased gastrointestinal length by the time of measurement at day 21. Embryonic leucine activates the brush borders enzymes such as leucine amino peptidase that facilitate nutrient digestion and absorption [42,43]. Additionally, the increased gastrointestinal length supports physiological adaptation and maintenance of intestinal homeostasis, ensuring nutrient absorption for survival and growth in poultry [26,44,45]. 

We investigated the impact of in ovo leucine supplementation on postnatal growth and development in Japanese quail in relation to the activity of the nutrient-sensing IGF-1/mTOR pathway. We found that a subtle increase in embryonic leucine had a long-lasting programming effect on postnatal growth, which was mediated through the IGF-1/mTOR pathway. 

Our study focused on messenger RNA (mRNA) changes that provide an early indication of the cellular responses to stimuli such as nutrients and growth factors [46]. These stimuli initiate the expression of specific genes [47], which may affect the mRNA levels without the corresponding change in the respective protein levels [48]. Similarly, due to temporal dynamics of gene expression changes in response to nutrients, early changes in mRNA can be a predictive measure of the subsequent changes in the protein levels accompanied by the activation of the IGF-1/mTOR pathway downstream molecules [49]. mRNA is an important early marker that reprograms a cell to respond to the specific signal and predicts a desired new phenotypic trait before becoming apparent at the protein level. Additional mRNA regulatory mechanisms, such as post-transcriptional regulation, translational efficiency, and protein degradation, link mRNA and the phenotype [46,50,51]. 

In response to the leucine treatment, we found altered hepatic gene expression patterns in the nutrient-sensing IGF-1/mTOR pathway already at hatching. This is intriguing because phenotypic differences in growth manifested only at day 3, 5, or 7 (for tarsus length, body mass, and head and wing length, respectively); thus, the genetic upregulation preceded enhanced growth by several days. These results corroborate the findings of our recent study [8], where we showed that a 2% increase in embryonic methionine also had a programmatic effect on the developmental trajectories of Japanese quails. The effects of leucine seem to be even stronger than those of methionine, as the phenotypic differences manifested earlier and affected more skeletal variables. Moreover, the upregulation of *IGF1* lasted until day 21, whereas for methionine treatment, at day 21, the relative expression of *IGF1* was already similar between the treatment and the control group [8]. Apart from the differences in amino acids used in the two studies, the current study also employed supplementation at a later stage (day 10 vs. day 0 for methionine). Further studies are needed to disentangle whether a more advanced stage of embryonic development is responsible for the stronger growth-promoting effects in the current study, or if leucine is a more potent activator of the IGF-1/mTOR pathway and, thus, phenotypic growth. 

Similar to *IGF1* and *IGF1R*, the expression of *mTOR* and one of its key downstream effectors, ribosomal protein S6 kinase 1 (*RPS6K1*), was also increased at hatching in response to leucine treatment. mTOR plays a crucial role in regulating protein synthesis and cell differentiation, proliferation, and growth in response to nutrients such as leucine and growth factors [52,53]. In the presence of nutrients such as leucine, it activates *mTOR* complex 1 (mTORC1), which then phosphorylates the downstream effectors such as S6K1 proteins [54,55]. However, unlike *IGF1*, the relative expression of *IGF1R*, *mTOR*, and *RPS6K1* returned to the level of controls by day 21. This pattern partly resembles the effects found for methionine (8), where initially upregulated *mTOR* expression disappeared by the end of the experiment. However, the current study shows the opposite pattern for *RPS6K1*, which was upregulated only on day 21 following a methionine treatment [8], whereas here, it was only affected at hatching. S6K1 is the downstream effector of the mTOR pathway, and its activation requires mTOR-mediated phosphorylation for protein synthesis, cell size, and cell growth [56,57,58]. The difference in the *RPS6K1* gene expression pattern between leucine and methionine indicated that specific amino acids may have distinct roles in influencing postnatal growth and development in Japanese quail. Consistent with our results, dietary leucine supplementation post-hatch in broiler chickens [25] and Zebrafish (*Danio rerio*) [59] increased activation of *mTOR* and *RPS6K1* genes in the breast muscles with increased age. However, dietary supplementation of leucine in broilers at the ages of 1 to 10, 11 to 21, and 22 to 35 days neither influenced the relative expression of *mTOR* and *RPS6K1* nor improved growth performance [18]. These results indicate that embryonic treatment may provide significant and lasting growth compared to postnatal supplementation.

Maintaining the balance between protein synthesis and degradation is crucial for growth and development. Protein synthesis is interlinked with the protein kinase *B/Akt* pathway, with the Akt/mTOR pathway stimulating protein synthesis, and the Akt/FOXO1 pathway responsible for protein degradation [18,19]. FOXO1, a transcriptional factor downstream of the protein kinase B/Akt pathway, regulates cell survival, growth, and development [60,61]. Under nutrient scarcity, FOXO1 triggers autophagic and proteasomal gene transcription, breaking down skeletal muscle protein for gluconeogenesis [62,63,64]. However, excessive autophagy and protein degradation can have negative impacts, such as cell death and growth reduction [61,65]. Our results showed that *FOXO1* gene expression did not show a difference between the leucine and controls in both day-old and 21-day-old chicks. The increase in the expression of the *FOXO1* gene reduces the accumulation of toxic protein in mammals [61], which suggests a positive influence on protein metabolism and improves the growth of poultry. For example, thermal manipulation elevated *FOXO1* gene expression during embryogenesis, accelerating broiler growth [66]. Dietary leucine supplementation in broiler chicks at ages 1 to 10, 11 to 21, and 22 to 35 did not show a difference in *FOXO1* gene expression [18]. However, the transcriptional analysis of FOXO genes showed increased transcriptomes of broilers compared to layers, suggesting that the FOXO genes, including the *FOXO1* gene, are also essential in regulating growth and metabolism in poultry [67]. Despite the contradictory evidence, this shows that the *FOXO1* gene is responsible for controlling growth in poultry.

## 5. Conclusions

Taken together, our study revealed that embryonic supplementation of a subtle dose of leucine triggers several key elements of the IIS/mTOR pathway and promotes enhanced postnatal growth in Japanese quail. Conducting comparative studies across various avian species and developmental stages will provide a broader perspective on how specific nutrients influence growth and development. Conducting a longitudinal study that examines multiple supplementation time points would clarify the optimal timing for maximising growth effects. Longitudinal studies tracking quail from hatching to adulthood would help assess whether early growth advantages translate into long-term benefits, such as improved reproductive performance, survivability, or overall health. Exploring the molecular mechanisms at different stages could shed light on why certain time points are more effective. Moreover, investigating the combined effects of leucine and other essential amino acids could potentially uncover synergistic influences on growth and development. These results highlight the potential biological importance of nutritional cues as a form of early maternal investment to adjust offspring phenotypes [68,69] while opening an avenue for enhancing poultry productivity.

## Figures and Tables

**Table 1:** Amino acid (m/m%) content of Japanese quail eggs. Concentrations were calculated from a pool of 12 freshly laid eggs.

Amino Acid	Whole Egg	Egg Yolk	Egg White
ASP	1.26	1.43	1.04
THR	0.73	0.82	0.60
SER	0.98	1.23	0.69
GLU	1.82	1.96	1.50
PRO	0.47	0.69	0.38
GLY	0.40	0.44	0.36
ALA	0.59	0.66	0.55
CYS	0.20	0.21	0.28
VAL	0.80	0.90	0.66
MET	0.42	0.38	0.36
ILE	0.65	0.77	0.50
LEU	1.11	1.20	0.85
TYR	0.54	0.65	0.42
PHE	0.68	0.68	0.64
HIS	0.36	0.44	0.27
LYS	1.18	1.20	0.87
ARG	0.58	0.80	0.37

**Table 2:** Dietary composition and calculated nutrient content of quail grower diet.

Feed Ingredients	%
Corn	23.69
Wheat	30.00
Soybean meal (46% CP)	34.85
Fishmeal	5.00
Sunflower oil	4.09
Limestone	1.01
MCP	0.37
Salt	0.24
DL-Methionine	0.10
L-Threonine	0.13
Vitamin and mineral premix ^a^	0.50
Nutrients	%
Metabolisable energy (MJ/kg)	12.13
Crude protein	24.00
Calcium	0.80
Available phosphorus	0.30
Sodium	0.15
Methionine	0.45
Lysine	1.34
Threonine	1.02
Tryptophan	0.29

^a^ 1 kg premix provided 1,000,000 NE vitamin A, 200,000 NE vitamin D_3_, 4900 mg/kg vitamin E, 200 mg vitamin K_3_, 150 mg vitamin B_1_, 500 mg vitamin B_2_, 1200 mg Ca-d-Pantothetane, 400 mg vitamin B_6_, 2 mg vitamin B_12_, 11 mg biotin, 2502 mg niacin, 60 mg folic acid, 300,000 mg choline chloride, 13200 mg Zn, 1920 mg Cu, 9612 mg Fe, 13,200 mg Mn, 180 mg I, 42 mg Se, and 12 mg Co.

**Table 3:** Primer sequence of the target genes (*IGF1*, *IGFR*, *mTOR*, *RPS6K1*, *FOXO1*) and reference gene (*RPL19*).

Gene	Gene Name	Primer Sequences (5′ → 3′) (Forward/Reverse)	NCBI GenBank	Amplicon Length Range (bp)
*mTOR*	Mechanistic target of rapamycin	F: CCGAAGCATTGAATTGGCCCT	XM_015882433.2	116
		R: CATCTCTCAAAGGCAGCGGACC		
*RPS6K1*	Ribosomal protein S6 kinase 1	F: AGGCAGGAACCCTCCGTGCAA	XM_015883670.2	106
		R: AGCTCAAACTGCGAAGGGTCGG		
*IGF-1*	Insulin-like growth factor-1	F: CACTATGCGGTGCTGAGCTGGTT	XM_015867574.2	118
		R: TCCCCTTGTGGTGTAAG CGTCT		
*IGF1R*	Insulin-like growth factor 1 receptor	F: TACAACTACCGCTGCTGGACCAC	XM_015873184.2	107
		R: AGGCACTCAGGATGGCAACAC		
*FOXO1*	Forkhead box O1	F: TGAGCGAGATCTGCGAGTTCAT	XM_015851898.1	102
		R: AGGAAGCTCCCGTTGTCGAACA		
*RPL19*	Ribosomal protein L19	F: CATCGGTAAGAGGAAGGGT	XM_015885843.1	163
		R: ACGTTGCCCTTGACCTTCAG		

**Table 4:** Estimates and *p*-values of the statistical models for body mass, head length, tarsus length, and wing length following embryonic leucine treatment. The estimates and the statistical values show pairwise comparisons of estimated marginal means (emmeans) in the leucine-treated and control groups at different ages of postnatal development.

Parameter	Day	Estimate	SE.	df	t-Ratio	Lower.CL	Upper.CL	*p*-Value
Body mass (g)	1	−0.67	2.56	61.60	−0.26	−5.79	4.44	0.795
	3	−5.70	3.50	91.80	−1.63	−12.64	1.25	0.107
	5	−9.10	3.75	98.44	−2.43	−16.55	−1.65	0.017
	7	−13.73	3.82	100.19	−3.59	−21.32	−6.14	<0.001
	10	−19.35	3.82	100.19	−5.06	−26.94	−11.77	<0.001
	14	−28.57	3.82	100.19	−7.47	−36.16	−20.98	<0.001
	21	−30.29	3.98	103.71	−7.62	−38.17	−22.40	<0.001
Head length (mm)	1	0.35	0.57	57.60	0.60	−0.80	1.49	0.550
	3	−0.80	0.78	89.65	−1.04	−2.34	0.74	0.303
	5	−1.63	0.83	96.80	−1.97	−3.27	0.01	0.052
	7	−3.19	0.84	98.68	−3.79	−4.86	−1.52	<0.001
	10	−3.07	0.84	98.68	−3.64	−4.74	−1.40	<0.001
	14	−3.78	0.84	98.68	−4.48	−5.45	−2.11	<0.001
	21	−3.88	0.87	102.43	−4.43	−5.61	−2.14	<0.001
Tarsus length (mm)	1	0.16	0.59	58.86	0.27	−1.03	1.35	0.789
	3	−2.10	0.81	90.37	−2.61	−3.70	−0.50	0.011
	5	−1.66	0.86	97.37	−1.93	−3.37	0.05	0.006
	7	−2.49	0.88	99.20	−2.84	−4.24	−0.75	0.005
	10	−3.31	0.88	99.20	−3.77	−5.05	−1.56	<0.001
	14	−4.30	0.88	99.20	−4.90	−6.04	−2.56	<0.001
	21	−4.11	0.91	102.88	−4.51	−5.92	−2.30	<0.001
Wing length (mm)	1	−0.07	1.81	86.45	−0.04	−3.67	3.53	0.969
	3	−0.67	2.58	101.05	−0.26	−5.79	4.46	0.797
	5	−3.16	2.80	104.40	−1.13	−8.72	2.40	0.263
	7	−6.46	2.87	105.37	−2.25	−12.15	−0.78	0.026
	10	−11.65	2.87	105.37	−4.06	−17.34	−5.97	<0.001
	14	−15.03	2.87	105.37	−5.24	−20.71	−9.34	<0.001
	21	−12.76	3.01	107.54	−4.24	−18.72	−6.80	<0.001

## Data Availability

Raw data and supporting analyses are submitted as Supplementary Materials.

## Supplementary Materials

The following supporting information can be downloaded at: https://www.mdpi.com/article/10.3390/ani14172596/s1, Table S1: Mean, standard deviation (SD) and standard error of the mean (SE) of body mass, head length, wing length and tarsal length of each day in each treatment.
