# Supplementary material for: Embryonic Leucine Promotes Early Postnatal Growth via mTOR Signalling in Japanese Quails
Source: Animals (Basel). 2024 Sep 6;14(17):2596. doi: 10.3390/ani14172596 (PMC11394045; doi:10.3390/ani14172596)
Supplement: Supplementary file 1 [file animals-14-02596-s001.zip › Anamals_Analysis-script.html]

Embryonic leucine promotes postnatal growth via mTOR signalling in Japanese quails


# Embryonic leucine promotes postnatal growth via mTOR signalling in Japanese quails

#### Sawadi

#### 09/20/2023

# Loading data

# Hatching success

We observed no difference in hatching success between the
leucine-injected (61.5%) and the control groups (50%) (ꭓ2 = 0.59741, df
= 1, p-value = 0.4396)

```
##          Outcome
## Group     Developed_Hatched Developed_Not_Hatched
##   Control                18                    18
##   Leucine                24                    15
```

```
## 
##  Pearson's Chi-squared test with Yates' continuity correction
## 
## data:  contingency_table2
## X-squared = 0.59741, df = 1, p-value = 0.4396
```

# Growth

## Body mass

### Fig1A

### statistics

```
## Linear mixed model fit by REML. t-tests use Satterthwaite's method [
## lmerModLmerTest]
## Formula: bodymass ~ treatment * as.factor(day) + (1 | birdID)
##    Data: growth
## 
## REML criterion at convergence: 758.6
## 
## Scaled residuals: 
##     Min      1Q  Median      3Q     Max 
## -3.0358 -0.3447  0.0113  0.2640  3.3696 
## 
## Random effects:
##  Groups   Name        Variance Std.Dev.
##  birdID   (Intercept) 27.07    5.203   
##  Residual             31.09    5.576   
## Number of obs: 124, groups:  birdID, 36
## 
## Fixed effects:
##                                   Estimate Std. Error       df t value Pr(>|t|)
## (Intercept)                         9.2125     1.9067  74.9777   4.832 7.00e-06
## treatmentLeucine                    0.6725     2.5580  74.9777   0.263  0.79335
## as.factor(day)3                     2.4152     2.7473 102.0207   0.879  0.38141
## as.factor(day)5                     7.1710     2.9195 102.1053   2.456  0.01573
## as.factor(day)7                    16.5877     2.9195 102.1053   5.682 1.27e-07
## as.factor(day)10                   21.6544     2.9195 102.1053   7.417 3.64e-11
## as.factor(day)14                   31.0377     2.9195 102.1053  10.631  < 2e-16
## as.factor(day)21                   53.9091     3.1152 101.0598  17.305  < 2e-16
## treatmentLeucine:as.factor(day)3    5.0237     3.5385 101.0083   1.420  0.15876
## treatmentLeucine:as.factor(day)5    8.4269     3.7847 101.2350   2.227  0.02819
## treatmentLeucine:as.factor(day)7   13.0589     3.8549 101.1215   3.388  0.00101
## treatmentLeucine:as.factor(day)10  18.6797     3.8549 101.1215   4.846 4.56e-06
## treatmentLeucine:as.factor(day)14  27.8964     3.8549 101.1215   7.237 9.13e-11
## treatmentLeucine:as.factor(day)21  29.6124     4.0052 100.5434   7.394 4.36e-11
##                                      
## (Intercept)                       ***
## treatmentLeucine                     
## as.factor(day)3                      
## as.factor(day)5                   *  
## as.factor(day)7                   ***
## as.factor(day)10                  ***
## as.factor(day)14                  ***
## as.factor(day)21                  ***
## treatmentLeucine:as.factor(day)3     
## treatmentLeucine:as.factor(day)5  *  
## treatmentLeucine:as.factor(day)7  ** 
## treatmentLeucine:as.factor(day)10 ***
## treatmentLeucine:as.factor(day)14 ***
## treatmentLeucine:as.factor(day)21 ***
## ---
## Signif. codes:  0 '***' 0.001 '**' 0.01 '*' 0.05 '.' 0.1 ' ' 1
```

Leucine injection on embryonic day 10 (after incubation) did not
affect body mass on day-old and three days old chicks after hatching (p=
> 0.05, figure….). However, five days later after hatching, body mass
increased significantly in leucine injected group than the control, the
effect that persisted until 21 days (p < 0.05, figure….).

## Head length

### Fig1B

### statistics

```
## Linear mixed model fit by REML. t-tests use Satterthwaite's method [
## lmerModLmerTest]
## Formula: headlength ~ treatment * as.factor(day) + (1 | birdID)
##    Data: growth
## 
## REML criterion at convergence: 423.9
## 
## Scaled residuals: 
##     Min      1Q  Median      3Q     Max 
## -4.9670 -0.3801  0.0521  0.4339  2.0499 
## 
## Random effects:
##  Groups   Name        Variance Std.Dev.
##  birdID   (Intercept) 1.489    1.220   
##  Residual             1.432    1.197   
## Number of obs: 124, groups:  birdID, 36
## 
## Fixed effects:
##                                   Estimate Std. Error      df t value Pr(>|t|)
## (Intercept)                        20.0750     0.4273 59.4387  46.983  < 2e-16
## treatmentLeucine                   -0.3450     0.5733 59.4387  -0.602 0.549584
## as.factor(day)3                     2.0577     0.5940 96.6633   3.464 0.000794
## as.factor(day)5                     3.4217     0.6312 96.6732   5.421 4.36e-07
## as.factor(day)7                     4.1217     0.6312 96.6732   6.530 3.06e-09
## as.factor(day)10                    7.7550     0.6312 96.6732  12.286  < 2e-16
## as.factor(day)14                    8.7217     0.6312 96.6732  13.817  < 2e-16
## as.factor(day)21                    9.7719     0.6731 95.1743  14.518  < 2e-16
## treatmentLeucine:as.factor(day)3    1.1465     0.7646 95.2575   1.500 0.137024
## treatmentLeucine:as.factor(day)5    1.9763     0.8178 95.4480   2.416 0.017576
## treatmentLeucine:as.factor(day)7    3.5357     0.8329 95.2631   4.245 5.08e-05
## treatmentLeucine:as.factor(day)10   3.4149     0.8329 95.2631   4.100 8.70e-05
## treatmentLeucine:as.factor(day)14   4.1232     0.8329 95.2631   4.950 3.20e-06
## treatmentLeucine:as.factor(day)21   4.2230     0.8651 94.4424   4.881 4.27e-06
##                                      
## (Intercept)                       ***
## treatmentLeucine                     
## as.factor(day)3                   ***
## as.factor(day)5                   ***
## as.factor(day)7                   ***
## as.factor(day)10                  ***
## as.factor(day)14                  ***
## as.factor(day)21                  ***
## treatmentLeucine:as.factor(day)3     
## treatmentLeucine:as.factor(day)5  *  
## treatmentLeucine:as.factor(day)7  ***
## treatmentLeucine:as.factor(day)10 ***
## treatmentLeucine:as.factor(day)14 ***
## treatmentLeucine:as.factor(day)21 ***
## ---
## Signif. codes:  0 '***' 0.001 '**' 0.01 '*' 0.05 '.' 0.1 ' ' 1
```

Leucine injection on embryonic day 10 (after incubation) did not
affect head length on day-old and three days old chicks after hatching
(p = > 0.05, figure….). However, five days later after hatching, head
length increased significantly in leucine injected group than the
control, the effect that persisted until 21 days (p < 0.05,
figure….).

## Tarsus length

### Fig1C

### statistics

```
## Linear mixed model fit by REML. t-tests use Satterthwaite's method [
## lmerModLmerTest]
## Formula: tarsuslength ~ treatment * as.factor(day) + (1 | birdID)
##    Data: growth
## 
## REML criterion at convergence: 433.6
## 
## Scaled residuals: 
##     Min      1Q  Median      3Q     Max 
## -3.1795 -0.4084 -0.0176  0.5402  2.2459 
## 
## Random effects:
##  Groups   Name        Variance Std.Dev.
##  birdID   (Intercept) 1.552    1.246   
##  Residual             1.583    1.258   
## Number of obs: 124, groups:  birdID, 36
## 
## Fixed effects:
##                                   Estimate Std. Error      df t value Pr(>|t|)
## (Intercept)                        16.1750     0.4426 63.1560  36.543  < 2e-16
## treatmentLeucine                   -0.1600     0.5939 63.1560  -0.269 0.788480
## as.factor(day)3                     1.6263     0.6229 98.0988   2.611 0.010445
## as.factor(day)5                     4.5615     0.6619 98.1411   6.891 5.36e-10
## as.factor(day)7                     4.9949     0.6619 98.1411   7.546 2.32e-11
## as.factor(day)10                    8.6949     0.6619 98.1411  13.136  < 2e-16
## as.factor(day)14                   10.5282     0.6619 98.1411  15.905  < 2e-16
## as.factor(day)21                   12.6882     0.7060 96.7358  17.972  < 2e-16
## treatmentLeucine:as.factor(day)3    2.2598     0.8019 96.7684   2.818 0.005859
## treatmentLeucine:as.factor(day)5    1.8224     0.8578 96.9862   2.124 0.036172
## treatmentLeucine:as.factor(day)7    2.6537     0.8736 96.8193   3.038 0.003066
## treatmentLeucine:as.factor(day)10   3.4662     0.8736 96.8193   3.968 0.000139
## treatmentLeucine:as.factor(day)14   4.4579     0.8736 96.8193   5.103 1.67e-06
## treatmentLeucine:as.factor(day)21   4.2729     0.9075 96.0480   4.709 8.40e-06
##                                      
## (Intercept)                       ***
## treatmentLeucine                     
## as.factor(day)3                   *  
## as.factor(day)5                   ***
## as.factor(day)7                   ***
## as.factor(day)10                  ***
## as.factor(day)14                  ***
## as.factor(day)21                  ***
## treatmentLeucine:as.factor(day)3  ** 
## treatmentLeucine:as.factor(day)5  *  
## treatmentLeucine:as.factor(day)7  ** 
## treatmentLeucine:as.factor(day)10 ***
## treatmentLeucine:as.factor(day)14 ***
## treatmentLeucine:as.factor(day)21 ***
## ---
## Signif. codes:  0 '***' 0.001 '**' 0.01 '*' 0.05 '.' 0.1 ' ' 1
```

Leucine injection on embryonic day 10 (after incubation) did not
affect tarsi length on day-old after hatching (p = > 0.05, figure….).
However, three days later, tarsi length increased significantly in
leucine injected group than the control, the effect that persisted until
21 days (p < 0.05, figure….).

## Wing length

### Fig1D

### statistics

```
## Linear mixed model fit by REML. t-tests use Satterthwaite's method [
## lmerModLmerTest]
## Formula: winglength ~ treatment * as.factor(day) + (1 | birdID)
##    Data: growth
## 
## REML criterion at convergence: 702.4
## 
## Scaled residuals: 
##     Min      1Q  Median      3Q     Max 
## -3.0287 -0.3277  0.0335  0.3230  3.3364 
## 
## Random effects:
##  Groups   Name        Variance Std.Dev.
##  birdID   (Intercept)  7.415   2.723   
##  Residual             21.749   4.664   
## Number of obs: 124, groups:  birdID, 36
## 
## Fixed effects:
##                                   Estimate Std. Error      df t value Pr(>|t|)
## (Intercept)                         15.250      1.350  95.588  11.295  < 2e-16
## treatmentLeucine                     0.070      1.811  95.588   0.039 0.969254
## as.factor(day)3                      1.878      2.216 105.184   0.848 0.398607
## as.factor(day)5                      7.296      2.352 105.684   3.101 0.002469
## as.factor(day)7                     10.096      2.352 105.684   4.292 3.94e-05
## as.factor(day)10                    23.629      2.352 105.684  10.045  < 2e-16
## as.factor(day)14                    30.979      2.352 105.684  13.169  < 2e-16
## as.factor(day)21                    42.235      2.518 105.022  16.774  < 2e-16
## treatmentLeucine:as.factor(day)3     0.597      2.864 104.245   0.208 0.835274
## treatmentLeucine:as.factor(day)5     3.086      3.058 104.949   1.009 0.315204
## treatmentLeucine:as.factor(day)7     6.390      3.115 104.977   2.051 0.042734
## treatmentLeucine:as.factor(day)10   11.582      3.115 104.977   3.718 0.000325
## treatmentLeucine:as.factor(day)14   14.957      3.115 104.977   4.801 5.26e-06
## treatmentLeucine:as.factor(day)21   12.689      3.242 104.615   3.914 0.000162
##                                      
## (Intercept)                       ***
## treatmentLeucine                     
## as.factor(day)3                      
## as.factor(day)5                   ** 
## as.factor(day)7                   ***
## as.factor(day)10                  ***
## as.factor(day)14                  ***
## as.factor(day)21                  ***
## treatmentLeucine:as.factor(day)3     
## treatmentLeucine:as.factor(day)5     
## treatmentLeucine:as.factor(day)7  *  
## treatmentLeucine:as.factor(day)10 ***
## treatmentLeucine:as.factor(day)14 ***
## treatmentLeucine:as.factor(day)21 ***
## ---
## Signif. codes:  0 '***' 0.001 '**' 0.01 '*' 0.05 '.' 0.1 ' ' 1
```

Leucine injection on embryonic day 10 (after incubation) did not
affect wing length on day-old up to five days old chicks after hatching
(p = > 0.05, figure….). However, seven days later, wing length
increased significantly in leucine injected group than the control, the
effect that persisted until 21 days when the experiment was terminated
(p < 0.05, figure….).

## Figure 1

##Intestinal length

```
## 
## Call:
## lm(formula = intestlength ~ treatment * as.factor(day), data = intestine)
## 
## Residuals:
##     Min      1Q  Median      3Q     Max 
## -10.938  -1.975  -0.175   1.825   9.863 
## 
## Coefficients:
##                                   Estimate Std. Error t value Pr(>|t|)    
## (Intercept)                         17.175      1.645  10.440 1.33e-10 ***
## treatmentLeucine                     1.500      2.326   0.645   0.5250    
## as.factor(day)21                    25.585      2.653   9.645 6.61e-10 ***
## treatmentLeucine:as.factor(day)21    8.677      3.528   2.459   0.0212 *  
## ---
## Signif. codes:  0 '***' 0.001 '**' 0.01 '*' 0.05 '.' 0.1 ' ' 1
## 
## Residual standard error: 4.653 on 25 degrees of freedom
##   (20 observations deleted due to missingness)
## Multiple R-squared:  0.9307, Adjusted R-squared:  0.9223 
## F-statistic: 111.8 on 3 and 25 DF,  p-value: 1.293e-14
```

# Gene expression

## Statistics

# IGF1 gene model

```
## 
## Call:
## lm(formula = IGF1 ~ treatment * day, data = genes)
## 
## Residuals:
##     Min      1Q  Median      3Q     Max 
## -5.2350 -1.0141  0.6767  1.3014  2.6080 
## 
## Coefficients:
##                      Estimate Std. Error t value Pr(>|t|)    
## (Intercept)           2.90015    0.76949   3.769 0.000895 ***
## treatmentLeucine      2.34976    1.08786   2.160 0.040559 *  
## day                  -0.04525    0.05898  -0.767 0.450084    
## treatmentLeucine:day  0.03033    0.07845   0.387 0.702306    
## ---
## Signif. codes:  0 '***' 0.001 '**' 0.01 '*' 0.05 '.' 0.1 ' ' 1
## 
## Residual standard error: 2.069 on 25 degrees of freedom
##   (10 observations deleted due to missingness)
## Multiple R-squared:  0.3209, Adjusted R-squared:  0.2394 
## F-statistic: 3.938 on 3 and 25 DF,  p-value: 0.01979
```

```
## 
## Call:
## lm(formula = IGF1 ~ treatment, data = data_day1)
## 
## Residuals:
##     Min      1Q  Median      3Q     Max 
## -5.2350 -1.1049  0.3682  0.9721  2.6080 
## 
## Coefficients:
##                  Estimate Std. Error t value Pr(>|t|)    
## (Intercept)        2.8549     0.6639   4.300 0.000733 ***
## treatmentLeucine   2.3801     0.9389   2.535 0.023795 *  
## ---
## Signif. codes:  0 '***' 0.001 '**' 0.01 '*' 0.05 '.' 0.1 ' ' 1
## 
## Residual standard error: 1.878 on 14 degrees of freedom
##   (10 observations deleted due to missingness)
## Multiple R-squared:  0.3146, Adjusted R-squared:  0.2657 
## F-statistic: 6.427 on 1 and 14 DF,  p-value: 0.02379
```

```
## 
## Call:
## lm(formula = IGF1 ~ treatment, data = data_day21)
## 
## Residuals:
##     Min      1Q  Median      3Q     Max 
## -4.9365 -0.7195  1.1736  1.5436  1.8182 
## 
## Coefficients:
##                  Estimate Std. Error t value Pr(>|t|)  
## (Intercept)         1.950      1.024   1.904   0.0833 .
## treatmentLeucine    2.987      1.305   2.288   0.0429 *
## ---
## Signif. codes:  0 '***' 0.001 '**' 0.01 '*' 0.05 '.' 0.1 ' ' 1
## 
## Residual standard error: 2.29 on 11 degrees of freedom
## Multiple R-squared:  0.3225, Adjusted R-squared:  0.2609 
## F-statistic: 5.236 on 1 and 11 DF,  p-value: 0.04291
```

#IGF1R gene model

```
## 
## Call:
## lm(formula = IGF1R ~ treatment * day, data = genes)
## 
## Residuals:
##     Min      1Q  Median      3Q     Max 
## -4.9723 -0.2537  0.1193  0.5726  3.1964 
## 
## Coefficients:
##                      Estimate Std. Error t value Pr(>|t|)    
## (Intercept)           5.18415    0.61552   8.422 2.49e-08 ***
## treatmentLeucine      2.68772    0.84281   3.189 0.004240 ** 
## day                  -0.21181    0.04534  -4.672 0.000117 ***
## treatmentLeucine:day -0.11689    0.06167  -1.895 0.071284 .  
## ---
## Signif. codes:  0 '***' 0.001 '**' 0.01 '*' 0.05 '.' 0.1 ' ' 1
## 
## Residual standard error: 1.549 on 22 degrees of freedom
##   (13 observations deleted due to missingness)
## Multiple R-squared:  0.8033, Adjusted R-squared:  0.7765 
## F-statistic: 29.94 on 3 and 22 DF,  p-value: 5.98e-08
```

```
## 
## Call:
## lm(formula = IGF1R ~ treatment, data = data_day1)
## 
## Residuals:
##     Min      1Q  Median      3Q     Max 
## -4.9723 -0.5306  0.2929  1.0255  3.1964 
## 
## Coefficients:
##                  Estimate Std. Error t value Pr(>|t|)    
## (Intercept)        4.9723     0.7468   6.658 1.57e-05 ***
## treatmentLeucine   2.5708     1.0226   2.514   0.0259 *  
## ---
## Signif. codes:  0 '***' 0.001 '**' 0.01 '*' 0.05 '.' 0.1 ' ' 1
## 
## Residual standard error: 1.976 on 13 degrees of freedom
##   (11 observations deleted due to missingness)
## Multiple R-squared:  0.3271, Adjusted R-squared:  0.2754 
## F-statistic:  6.32 on 1 and 13 DF,  p-value: 0.0259
```

```
## 
## Call:
## lm(formula = IGF1R ~ treatment, data = data_day21)
## 
## Residuals:
##      Min       1Q   Median       3Q      Max 
## -0.73612 -0.24618 -0.03604  0.32047  0.67097 
## 
## Coefficients:
##                  Estimate Std. Error t value Pr(>|t|)   
## (Intercept)        0.7361     0.2109   3.491  0.00682 **
## treatmentLeucine   0.2331     0.2855   0.816  0.43534   
## ---
## Signif. codes:  0 '***' 0.001 '**' 0.01 '*' 0.05 '.' 0.1 ' ' 1
## 
## Residual standard error: 0.4716 on 9 degrees of freedom
##   (2 observations deleted due to missingness)
## Multiple R-squared:  0.06895,    Adjusted R-squared:  -0.0345 
## F-statistic: 0.6665 on 1 and 9 DF,  p-value: 0.4353
```

#mTOR gene model

```
## 
## Call:
## lm(formula = mTOR ~ treatment * day, data = genes)
## 
## Residuals:
##     Min      1Q  Median      3Q     Max 
## -2.1141 -0.7044 -0.2937  0.9470  2.2042 
## 
## Coefficients:
##                      Estimate Std. Error t value Pr(>|t|)   
## (Intercept)           0.96785    0.46159   2.097  0.04629 * 
## treatmentLeucine      2.03456    0.65257   3.118  0.00454 **
## day                   0.06234    0.03538   1.762  0.09028 . 
## treatmentLeucine:day -0.11541    0.04706  -2.452  0.02151 * 
## ---
## Signif. codes:  0 '***' 0.001 '**' 0.01 '*' 0.05 '.' 0.1 ' ' 1
## 
## Residual standard error: 1.241 on 25 degrees of freedom
##   (10 observations deleted due to missingness)
## Multiple R-squared:  0.2832, Adjusted R-squared:  0.1972 
## F-statistic: 3.292 on 3 and 25 DF,  p-value: 0.03702
```

```
## 
## Call:
## lm(formula = mTOR ~ treatment, data = data_day1)
## 
## Residuals:
##     Min      1Q  Median      3Q     Max 
## -2.1141 -0.7018 -0.3353  0.5127  2.2042 
## 
## Coefficients:
##                  Estimate Std. Error t value Pr(>|t|)   
## (Intercept)        1.0302     0.4513   2.283  0.03858 * 
## treatmentLeucine   1.9192     0.6382   3.007  0.00942 **
## ---
## Signif. codes:  0 '***' 0.001 '**' 0.01 '*' 0.05 '.' 0.1 ' ' 1
## 
## Residual standard error: 1.276 on 14 degrees of freedom
##   (10 observations deleted due to missingness)
## Multiple R-squared:  0.3924, Adjusted R-squared:  0.349 
## F-statistic: 9.043 on 1 and 14 DF,  p-value: 0.009417
```

```
## 
## Call:
## lm(formula = mTOR ~ treatment, data = data_day21)
## 
## Residuals:
##     Min      1Q  Median      3Q     Max 
## -1.8880 -0.8075 -0.2937  0.9470  1.6393 
## 
## Coefficients:
##                  Estimate Std. Error t value Pr(>|t|)   
## (Intercept)        2.2770     0.5343   4.261  0.00134 **
## treatmentLeucine  -0.3890     0.6811  -0.571  0.57939   
## ---
## Signif. codes:  0 '***' 0.001 '**' 0.01 '*' 0.05 '.' 0.1 ' ' 1
## 
## Residual standard error: 1.195 on 11 degrees of freedom
## Multiple R-squared:  0.0288, Adjusted R-squared:  -0.05949 
## F-statistic: 0.3262 on 1 and 11 DF,  p-value: 0.5794
```

#RPS6K1 gene model

```
## 
## Call:
## lm(formula = RPS6K1 ~ treatment * day, data = genes)
## 
## Residuals:
##      Min       1Q   Median       3Q      Max 
## -1.21431 -0.33529 -0.06872  0.22200  2.07814 
## 
## Coefficients:
##                      Estimate Std. Error t value Pr(>|t|)  
## (Intercept)           0.44781    0.28495   1.572   0.1292  
## treatmentLeucine      0.77144    0.39010   1.978   0.0596 .
## day                   0.03343    0.02099   1.593   0.1242  
## treatmentLeucine:day -0.03837    0.02760  -1.390   0.1772  
## ---
## Signif. codes:  0 '***' 0.001 '**' 0.01 '*' 0.05 '.' 0.1 ' ' 1
## 
## Residual standard error: 0.7169 on 24 degrees of freedom
##   (11 observations deleted due to missingness)
## Multiple R-squared:  0.1668, Adjusted R-squared:  0.0626 
## F-statistic: 1.601 on 3 and 24 DF,  p-value: 0.2153
```

```
## 
## Call:
## lm(formula = RPS6K1 ~ treatment, data = data_day1)
## 
## Residuals:
##      Min       1Q   Median       3Q      Max 
## -1.21431 -0.28979 -0.08268  0.06288  2.07814 
## 
## Coefficients:
##                  Estimate Std. Error t value Pr(>|t|)  
## (Intercept)        0.4812     0.2809   1.713    0.110  
## treatmentLeucine   0.7331     0.3846   1.906    0.079 .
## ---
## Signif. codes:  0 '***' 0.001 '**' 0.01 '*' 0.05 '.' 0.1 ' ' 1
## 
## Residual standard error: 0.7432 on 13 degrees of freedom
##   (11 observations deleted due to missingness)
## Multiple R-squared:  0.2184, Adjusted R-squared:  0.1583 
## F-statistic: 3.633 on 1 and 13 DF,  p-value: 0.07901
```

```
## 
## Call:
## lm(formula = RPS6K1 ~ treatment, data = data_day21)
## 
## Residuals:
##      Min       1Q   Median       3Q      Max 
## -1.11553 -0.39119 -0.05476  0.68665  0.98470 
## 
## Coefficients:
##                  Estimate Std. Error t value Pr(>|t|)   
## (Intercept)        1.1499     0.3061   3.757  0.00317 **
## treatmentLeucine  -0.0344     0.3902  -0.088  0.93134   
## ---
## Signif. codes:  0 '***' 0.001 '**' 0.01 '*' 0.05 '.' 0.1 ' ' 1
## 
## Residual standard error: 0.6845 on 11 degrees of freedom
## Multiple R-squared:  0.0007059,  Adjusted R-squared:  -0.09014 
## F-statistic: 0.00777 on 1 and 11 DF,  p-value: 0.9313
```

#FOXO1 gene model

```
## 
## Call:
## lm(formula = FOXO1 ~ treatment * day, data = genes)
## 
## Residuals:
##      Min       1Q   Median       3Q      Max 
## -0.71533 -0.21747 -0.05242  0.18022  0.83732 
## 
## Coefficients:
##                      Estimate Std. Error t value Pr(>|t|)    
## (Intercept)          0.597424   0.157433   3.795 0.000884 ***
## treatmentLeucine     0.069102   0.230358   0.300 0.766777    
## day                  0.005614   0.012066   0.465 0.645911    
## treatmentLeucine:day 0.007144   0.016297   0.438 0.665057    
## ---
## Signif. codes:  0 '***' 0.001 '**' 0.01 '*' 0.05 '.' 0.1 ' ' 1
## 
## Residual standard error: 0.4233 on 24 degrees of freedom
##   (11 observations deleted due to missingness)
## Multiple R-squared:  0.1006, Adjusted R-squared:  -0.01181 
## F-statistic: 0.895 on 3 and 24 DF,  p-value: 0.4581
```

```
## 
## Call:
## lm(formula = FOXO1 ~ treatment, data = data_day1)
## 
## Residuals:
##      Min       1Q   Median       3Q      Max 
## -0.67928 -0.18195 -0.01386  0.15249  0.76246 
## 
## Coefficients:
##                  Estimate Std. Error t value Pr(>|t|)    
## (Intercept)       0.60304    0.13360   4.514 0.000583 ***
## treatmentLeucine  0.07625    0.19557   0.390 0.702953    
## ---
## Signif. codes:  0 '***' 0.001 '**' 0.01 '*' 0.05 '.' 0.1 ' ' 1
## 
## Residual standard error: 0.3779 on 13 degrees of freedom
##   (11 observations deleted due to missingness)
## Multiple R-squared:  0.01156,    Adjusted R-squared:  -0.06448 
## F-statistic: 0.152 on 1 and 13 DF,  p-value: 0.703
```

```
## 
## Call:
## lm(formula = FOXO1 ~ treatment, data = data_day21)
## 
## Residuals:
##      Min       1Q   Median       3Q      Max 
## -0.71533 -0.25516 -0.06802  0.35827  0.83732 
## 
## Coefficients:
##                  Estimate Std. Error t value Pr(>|t|)   
## (Intercept)        0.7153     0.2108   3.393    0.006 **
## treatmentLeucine   0.2191     0.2687   0.815    0.432   
## ---
## Signif. codes:  0 '***' 0.001 '**' 0.01 '*' 0.05 '.' 0.1 ' ' 1
## 
## Residual standard error: 0.4714 on 11 degrees of freedom
## Multiple R-squared:  0.05699,    Adjusted R-squared:  -0.02873 
## F-statistic: 0.6648 on 1 and 11 DF,  p-value: 0.4322
```

## Combined plots of gene expression

## Combined the gene expression plots
