# Supplementary material for: Embryonic Leucine Promotes Early Postnatal Growth via mTOR Signalling in Japanese Quails
Source: Animals (Basel). 2024 Sep 6;14(17):2596. doi: 10.3390/ani14172596 (PMC11394045; doi:10.3390/ani14172596)
Supplement: Supplementary file 1 [file animals-14-02596-s001.zip › Table S1.pdf]

Embryonic Leucine Promotes Postnatal Growth via mTOR Signalling in Japanese Quails

Sawadi F. Ndunguru[1,2,3], Gebrehaweria K. Reda[1,2,3], Brigitta Csernus[3], Renáta Knop[1], James K. Lugata[2,4], Csaba Szabó[4*], Ádám Z. Lendvai[3,†] and Levente Czeglédi[1,†]

[1]Department of Animal Science, Institute of Animal Science, Biotechnology and Nature Conservation, Faculty of Agricultural and Food Sciences and Environmental Management, University of Debrecen, 4032 Debrecen, Hungary

[2]Doctoral School of Animal Science, University of Debrecen, 4032 Debrecen, Hungary;

[3]Department of Evolutionary Zoology and Human Biology, University of Debrecen, 4032 Debrecen, Hungary

[4]Department of Animal Nutrition and Physiology, Institute of Animal Science, Biotechnology and Nature Conservation, Faculty of Agriculture and Food Sciences and Environmental Management, University of Debrecen, 4032 Debrecen, Hungary

* Correspondence: szabo.csaba@agr.unideb.hu

† These authors contributed equally to this work.

Table S1. Mean, standard deviation (SD) and standard error of the mean (**SE**) of body mass, head length, wing length and tarsal length of each day in each treatment

	**treament**	**day**	**Mean**	**Sample_Size (n)**	**SD**	**SE**
**Body mass (g)**	Control	1	9.21	16	1.46	0.37
	Control	3	11.24	7	3.17	1.20
	Control	5	16.12	6	6.69	2.73
	Control	7	25.53	6	9.93	4.05
	Control	10	30.60	6	12.86	5.25
	Control	14	39.98	6	17.49	7.14
	Control	21	64.54	5	20.27	9.06
	Leucine	1	9.89	20	1.14	0.26
	Leucine	3	17.56	11	3.86	1.16
	Leucine	5	26.20	9	5.86	1.95
	Leucine	7	40.44	8	6.68	2.36
	Leucine	10	51.13	8	8.05	2.85
	Leucine	14	69.73	8	10.77	3.81

	Leucine		94.31	8	12.40	4.38
**Head length (mm)**	Control	1	20.08	16	1.12	0.28
	Control	3	22.39	7	1.27	0.48
	Control	5	23.97	6	1.23	0.50
	Control	7	24.67	6	1.51	0.61
	Control	10	28.30	6	2.35	0.96
	Control	14	29.27	6	3.96	1.62
	Control	21	30.36	5	1.34	0.60
	Leucine	1	19.73	20	1.21	0.27
	Leucine	3	23.12	11	2.36	0.71
	Leucine	5	25.79	9	1.26	0.42
	Leucine	7	28.19	8	1.99	0.70
	Leucine	10	31.70	8	1.56	0.55
	Leucine	14	33.38	8	0.89	0.32
	Leucine	21	34.53	8	0.96	0.34
**Wing length (mm)**	Control	1	15.25	16	1.20	0.30
	Control	3	17.04	7	1.40	0.53
	Control	5	22.53	6	4.09	1.67
	Control	7	25.33	6	6.25	2.55
	Control	10	38.87	6	6.05	2.47
	Control	14	46.22	6	12.61	5.15
	Control	21	57.68	5	13.99	6.25
	Leucine	1	15.32	20	0.97	0.22
	Leucine	3	17.84	11	1.58	0.48
	Leucine	5	25.93	9	1.47	0.49
	Leucine	7	32.06	8	3.40	1.20
	Leucine	10	50.79	8	6.30	2.23
	Leucine	14	61.51	8	8.36	2.96
	Leucine	21	70.50	8	8.23	2.91
**Tarsus length (mm)**	Control	1	16.18	16	0.89	0.22
	Control	3	17.76	7	0.90	0.34
	Control	5	20.73	6	1.78	0.73
	Control	7	21.17	6	2.14	0.87
	Control	10	24.87	6	3.39	1.38
	Control	14	26.70	6	4.03	1.65
	Control	21	29.20	5	2.34	1.05
	Leucine	1	16.02	20	1.28	0.29
	Leucine	3	20.18	11	2.45	0.74
	Leucine	5	23.10	9	0.90	0.30
	Leucine	7	24.39	8	1.34	0.47

Leucine	10	28.90	8	1.00	0.36
Leucine	14	31.73	8	0.94	0.33
Leucine	21	33.70	8	1.65	0.58
